# Research Progress on PARP14 as a Drug Target

**DOI:** 10.3389/fphar.2019.00172

**Published:** 2019-03-05

**Authors:** Wei Qin, Hong-Jie Wu, Lu-Qi Cao, Hui-Jin Li, Chun-Xia He, Dong Zhao, Lu Xing, Peng-Quan Li, Xi Jin, Hui-Ling Cao

**Affiliations:** Shaanxi Key Laboratory of Ischemic Cardiovascular Disease, Shaanxi Key Laboratory of Brain disorders, Institute of Basic and Translational Medicine, Xi'an Medical University, Xi'an, China

**Keywords:** PARP14, drug target, cancer, atherosclerosis, allergic inflammation, molecular mechanism

## Abstract

Poly-adenosine diphosphate-ribose polymerase (PARP) implements posttranslational mono- or poly-ADP-ribosylation modification of target proteins. Among the known 18 members in the enormous family of PARP enzymes, several investigations about PARP1, PARP2, and PARP5a/5b have been launched in the past few decades; more specifically, PARP14 is gradually emerging as a promising drug target. An intact PARP14 (also named ARTD8 or BAL2) is constructed by macro1, macro2, macro3, WWE, and the catalytic domain. PARP14 takes advantage of nicotinamide adenine dinucleotide (NAD^+^) as a metabolic substrate to conduct mono-ADP-ribosylation modification on target proteins, taking part in cellular responses and signaling pathways in the immune system. Therefore, PARP14 has been considered a fascinating target for treatment of tumors and allergic inflammation. More importantly, PARP14 could be a potential target for a chemosensitizer based on the theory of synthetic lethality and its unique role in homologous recombination DNA repair. This review first gives a brief introduction on several representative PARP members. Subsequently, current literatures are presented to reveal the molecular mechanisms of PARP14 as a novel drug target for cancers (e.g., diffuse large B-cell lymphoma, multiple myeloma, prostate cancer, and hepatocellular carcinoma) and allergic inflammatory. Finally, potential PARP inhibitor-associated adverse effects are discussed. The review could be a meaningful reference for innovative drug or chemosensitizer discovery targeting to PARP14.

## Introduction

The poly-adenosine diphosphate-ribose polymerase (PARP) enzyme family catalyzes protein posttranslational ribosylation modification and utilizes nicotinamide adenine dinucleotide (NAD^+^) as a substrate to perform mono- or poly-ADP-ribosylation modification on target proteins (Amé et al., 2004; Vyas et al., 2014). Notably, the PARP family is also known as the ADP-ribosyltransferase Diphtheria-toxin like (ARTD) family, which is based on its enzymatic reaction and structural features (Hottiger et al., 2010). Another alias that describes some of its members is B-Aggressive lymphoma (BAL) proteins, in that some of PARP candidates are homologous with BAL proteins that were encoded by genes that were first discovered in diffuse large B-cell lymphoma (DLBCL) (Aguiar et al., 2005). For simplicity, this review will refer to the traditional name of PARP family.

PARP was first identified in 1963 (Chambon et al., 1963) and currently there are 18 members classified in different subgroups of the PARP family (Vyas et al., 2013). Interestingly, there is low amino acid sequence homology among its family members. Their N-termini have obvious variability in their domains and functional motifs, including regulatory motifs, the zinc finger, and ubiquitin-binding domains. However, most candidates have a similar conserved C-terminal catalytic domain. Representative members such as PARP1, PARP2, and PARP5a/5b have been investigated intensively, whereas studies on PARP14 are currently rare. As a mono-ADP-ribosyltransferase, PARP14 (ARTD8/BAL2) includes 1,801 amino acids and it mainly consists of macro1-3, WWE and the catalytic domain. Except the self-evident importance of the catalytic domain, the unique macro domains can engage in biological metabolism through binding mono (ADP-ribose). PARP14 can modify mono-ADP-ribosylation on target proteins, and thus elicit cellular responses. In fact, interest in PARP14 has grown in recent years as a potential drug target for tumors and allergic inflammation.

Because of its functions in ADP-ribosylation, PARP plays a key role in different processes of metabolism, such as DNA repair, transcription regulation and even pathogenesis of diseases. Remarkably, in tumor cells, PARP inhibitors can treat cancer by aggravating DNA damage from error-prone repair, inducing unstable genome that leads to the programmed death of cancer cells. Therefore, PARP has been a promising anticancer drug target for breast, ovarian, and prostate cancers. Many inhibitors in preclinical and clinical trails target both PARP1 and PARP2. In the Protein Data Bank (PDB), there are now 43 PARP1 or PARP1/2 inhibitors being released (http://www.rcsb.org/pdb/, 2019.01.31). Among them, a dozen PARP1/2 inhibitors are in clinical trials and four inhibitors (olaparib, rucaparib, niraparib, and talazoparib) have been approved by the US food and drug administration (FDA) for the treatment of breast cancer and ovarian cancer with breast cancer susceptibility gene 1/2 (*BRCA1/2*) genetic deficiency, fallopian tube carcinoma and peritoneal carcinoma (Gunderson and Moore, 2015; Dockery et al., 2017; Scott, 2017; Hoy, 2018).

Although more and more novel inhibitors are being evaluated in preclinical and clinical trials, there are two possible issues of vital concern in the application of PARP as a drug target. The first is the possibility of these inhibitors to function while bypassing PARP. For instance, minocycline, a potent PARP1 inhibitor, can protect against allergen-induced asthma by the means of controlling the T cell receptor (TCR)-nuclear factor κB (NF-κB)-trans-acting T-cell-specific transcription factor (GATA3)-interleukin-4 (IL-4) axis, without a direct modulation of PARP1 activity (Naura et al., 2013). Another thought-provoking proposal is the repurposing of PARP inhibitor such as olaparib for the treatment of other kinds of tumors beyond BRCA-deficient cancers. There is evidence that PARP1 regulates estrogen-dependent breast cancer cell growth through the modulation of the estrogen receptor (ER)-insulin-like growth factor 1 receptor (IGF-1R)-Na(+)/H(+) exchange regulatory cofactor NHE-RF3 (PDZK1) axis. Thus, PARP inhibitors may be powerful weapons for the therapy of ER-positive cancers (Kim et al., 2015).

As we briefly introduce the PARP family, highlight the number of recent literatures that elucidate the molecular mechanisms of PARP14 as a drug target for cancers and immunological diseases, and detail the potential toxicity for consideration, we hope this review becomes a significant reference in the development of a new drug or a chemosensitizer and further research concerning PARP14.

## Structure and Function of the PARP Members

PARPs perform posttranslational mono-/poly-ADP-ribosylation modification on target proteins, which include PARPs themselves (Amé et al., 2004). The negatively charged linear or branched chains of poly (ADP-ribose) on target proteins change the biochemical properties of these macromolecules, leading to the alteration of their structures and functions (Gagne et al., 2008). Activated PARP breaks down NAD^+^ into nicotinamide and ADP-ribose, and connects 50–200 ADP-ribose units to the target proteins via covalent bonds (Altmeyer et al., 2009). At sites of DNA damage, poly-ADP ribosylation PARP recruits various enzymes to initiate DNA repair, and to promote the growth and proliferation of cells (Satoh and Lindahl, 1992). Notably, the continuous activities of poly-ADP ribosylation may block rebinding of PARP to damaged DNA fragments, resulting in a pause of the repair process. To eliminate unintended effects, poly (ADP-ribose) glycohydrolase (PARG) cleaves the long poly (ADP-ribose) chains into short chains to recover the DNA damage repair activity of PARP (Satoh et al., 1994).

PARP1 and PARP2 have been amply studied for many years. PARP1 consists of three domains, including a DNA-binding domain, an auto-modification domain and a catalytic domain (Kameshita et al., 1984; Thomas and Tulin, 2013). The DNA-binding domain is composed of three zinc finger motifs involved in the recognition of damaged DNA regions. In addition, it contains a signal to facilitate PARP1 localization in the nucleus (Decker et al., 2000). When a catalytic process is completed, the auto-modification domain is responsible for protein dissociation from DNA substrate via the posttranslational modification on PARP1 (Altmeyer et al., 2009). Furthermore, a specific region within this domain, called the breast cancer-associated 1 C-terminal domain (BRCT), may mediate the binding activity between PARP1 and other partner molecules (Masson et al., 1998). The catalytic domain binds NAD^+^ to achieve poly-ADP ribosylation on target proteins, which is also the target of drug design (Langelier et al., 2011). PARP2 and PARP1 have similar structures, with 69% homology. The main difference is that PARP2 lacks an N-terminal DNA-binding domain. It has been speculated that several residues in the N-terminal domain of PARP2 may be capable of binding target DNA fragments and are involved in nuclear localization activity (Oliver et al., 2004). Due to their indispensable role in DNA repair and cellular metabolism, PARP1 and PARP2 are attractive anticancer targets. For instance, as the first PARP inhibitor, olaparib (Lynparza) was approved by the US FDA in 2014 as monotherapy for BRCA-mutant advanced ovarian cancer. In December 2016, the US FDA granted accelerated approval of rucaparib (Rubraca) for the therapy of previously treated BRCA-deficient ovarian cancer. Only 1 year later, niraparib (Zejula) was approved by the US FDA for the treatment of epithelial ovarian, fallopian tube, and primary peritoneal cancer. In October 2018, talazoparib (Talzenna) was approved for use as an oral PARP inhibitor by the US FDA for the treatment of BRCA-deficient, human epidermal growth factor receptor 2 (HER2)-negative, locally advanced or metastatic breast cancer (Gunderson and Moore, 2015; Dockery et al., 2017; Scott, 2017; Hoy, 2018).

PARP5a and PARP5b, also known as tankyrase 1 and tankyrase 2, may be other potential drug targets. There is a high level of homology between PARP5b and PARP5a (~85%), with the notable discrepancy being that there is no His-Pro-Ser motif in PARP5b. PARP5a/5b comprises of an ankyrin repeat domain, an oligomerization domain, and a catalytic domain (Morrone et al., 2012). The N-terminal of PARP5a contains a His-Pro-Ser motif, which has functional similarity to mitogen-activated protein kinase (MAPK). The enzymatic activity of PARP5a is markedly enhanced by the phosphorylation of this motif, activated by insulin stimulation, which indicates that PARP5a is a potential target in insulin signal transduction (De Rycker et al., 2003). Moreover, in the C-terminal region of PARP5a, the oligomerization domain (known as the sterile alpha motif, SAM) plays a crucial role during the process of PARP oligomerization and interaction with other target proteins (DaRosa et al., 2016).

**Figure 1:**
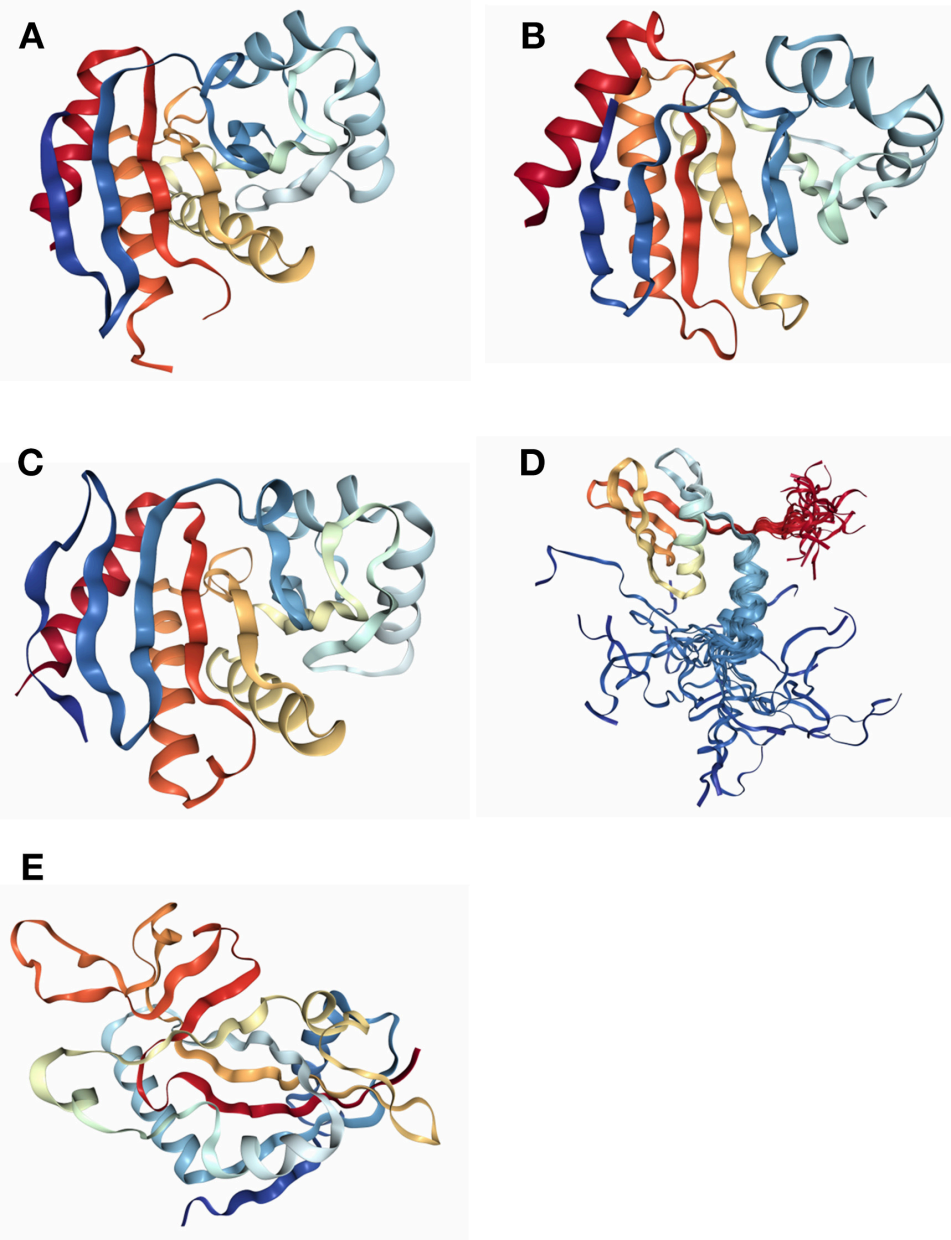
The determined domain structures of PARP14. **(A)** Crystal structure of macro domain 1 (PDB ID: 3Q6Z). **(B)** Crystal structure of macro domain 2 (PDB ID: 5O2D). **(C)** Crystal structure of macro domain 3 (PDB ID: 4ABL). **(D)** Solution structure of WWE domain (PDB ID: 1X4R). **(E)** Crystal structure of catalytic domain (PDB ID: 3SMJ).

Compared with PARP1, the domains of PARP14 are relatively simple. Full-length PARP14 is composed of five primary domains: macro1 (791–978), macro2 (1003–1190), macro3 (1216–1387), WWE (1523–1601), and a catalytic domain (1605–1801) (Forst et al., 2013). There are two contiguous RNA recognition motif (RRM) domains in front of the N-terminus, required for RNA recognition (Schweiker et al., 2018). From the determined crystal structures and solution structure (WWE) of these domains, it has been found that all three macro domains are very similar to some extent, with several β-sheets and 2-3 α-helixes on each side (Figure 1). More importantly, three consecutive macro domains (containing 180 amino acids) are engaged in ADP-ribose binding on the N-terminus, which can specifically recognize mono (ADP-ribose) rather than poly (ADP-ribose) (Feijs et al., 2013; Forst et al., 2013). Based on this function of macro domains, cofactor proteins might be linked by this bridge to form complicated functional complexes in biological metabolism. The WWE domain is particularly involved in protein-protein interactions in ubiquitination and is composed of three conserved residues (Trp-Trp-Glu) between the macro domains and the C-terminal catalytic domain (Aravind, 2001). The catalytic domain is involved in the mono-ADP-ribosylation modification of target proteins, which include PARP14 itself. Intriguingly, on account of special macro and catalytic domains, PARP14 is integrated with an executor, as well as a reader of ADP-ribosylation at the same time. In 2012, the first complex crystal structure of the catalytic domain and its inhibitor, 3-aminobenzamide (3AB), was determined, which laid the structural basis for drug discovery associated with this domain (Wahlberg et al., 2012). Plenty of literature indicated that PARP14 is not only related to cancers but also to other diseases, such as atherosclerosis and allergic inflammation. As a result, PARP14 has great potential as a new drug target for the treatment of diverse diseases.

## The Classical Mechanism of PARP14 as a Drug Target

PARP14 can cleave one NAD^+^ molecule into one ADP ribose and nicotinamide, and then transfer the mono (ADP-ribose) unit onto target proteins. Based on this particular biochemical reaction in cells, PARP14 has been regarded as an arrestive drug target for anticancer and anti-inflammatory therapy. But questions remain on how to achieve this intention and regarding the exact molecular mechanism behind this hypothesis. Until recent years, only one convincing mechanism has been discovered to illustrate the critical role of PARP14 in cellular signal pathway.

**Figure 2:**
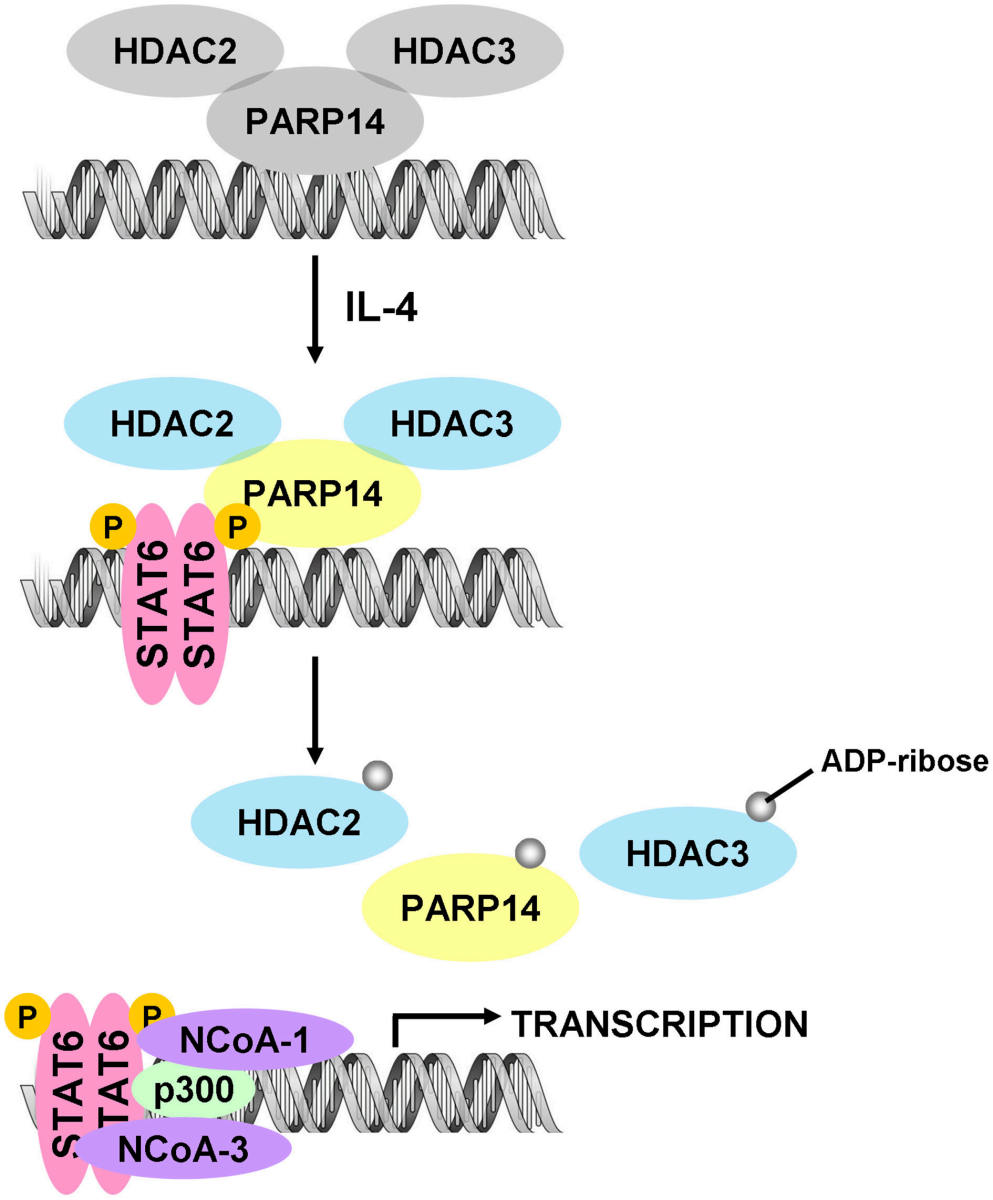
Diagram of classic molecular mechanism of PARP14 as a drug target. PARP14 acts as the molecular switch that controls IL-4 dependent gene transcription. In the absence of IL-4, PARP14, with HDAC2 and HDAC3 complex, binds the gene promoter to silence the gene. Subsequently, with IL-4 occurrence, STAT6 is activated and binds the specific promoter, leading to the mono-ADP ribosylation of PARP14, HDAC2, and HDAC3. This facilitates the dissociation of PARP14 and HDACs complex from the promoter, activating transcription co-factors such as p300, NCoA-1 and NCoA-3 to bind to the promoter, effectively initiating gene transcription (Mehrotra et al., 2011).

Signal transducer and activator of transcription 6 (STAT6) plays a key positive role in the regulation of IL-4-dependent gene activation. PARP14 acts as a molecular switch that controls gene transcription, beginning with STAT6-mediated IL-4 dependent gene transcription activation (Goenka et al., 2007; Mehrotra et al., 2011). At first, PARP14 was deemed a collaborator to STAT6 (CoaSt6) because of its ability to facilitate IL-4-dependent transcription, and the macro domains of PARP14 could enhance IL-4-induced gene expression by cooperation with STAT6 (Goenka and Boothby, 2006). In the absence of IL-4, PARP14 initially binds histone deacetylases 2 and 3 (HDAC2 and HDAC3), as well as IL-4 responsive promoters, to maintain gene silence. Upon IL-4 stimulation, STAT6 is activated to bind its target genes, which induces the catalytic activation of PARP14. Active PARP14 accomplishes mono-ADP-ribosylation on HDAC2, HDAC3 and itself, which allows PARP14 and HDACs to dissociate from IL-4-responsive promoters, in order to recruit nuclear receptor coactivator 1 and 3 (NCoA1 and NCoA3) and p300 to bind IL-4-responsive promoters. Next, histones are acetylated, and this starts a specific downstream gene transcription (Goenka and Boothby, 2006; Goenka et al., 2007; Mehrotra et al., 2011), as shown in Figure 2. Both the inactive mutant of PARP14 and inhibition of its activity using inhibitor 3AB block IL-4-dependent transcription from target promoters *in vivo* (Goenka et al., 2007).

In some tumor cells, cytokine IL-4 can act as a pro-survival signal factor and activate downstream STAT6 to begin responsive gene transcription for anti-apoptotic activity. Beyond the IL-4-STAT6 signaling pathway, there are some distinct and more complicated modes of communication among PARP14 and other cell factors implicated in physiological metabolism, exemplifying the complexity of disease pathogenesis. PARP14 could be a profound drug target as more knowledge beyond its classical mechanism is revealed.

## New Drug Target PARP14

### New Drug Target for Cancer

According to the classical mechanism mentioned above, as the transcriptional switch for STAT6-dependent gene expression, PARP14 takes advantage of mono-ADP-ribosyltransferase activity to mediate transcription of target genes involved in growth and proliferation of tumor cells (Goenka and Boothby, 2006; Goenka et al., 2007; Mehrotra et al., 2011). PARP14 could be a possible therapeutic target for cancers such as DLBCL. However, the pathogenesis of other types of cancers including multiple myeloma (MM), prostate cancer (PCa) and hepatocellular carcinoma (HCC) are not consistent with the classical mechanism. PARP14 would perform intricate roles in the development and progression of these tumors.

#### New Drug Target for Diffuse Large B-Cell Lymphoma (DLBCL)

DLBCL is a lymphoid malignancy of B cells, which are responsible for producing antibodies. As an aggressive tumor, DLBCL can arise in any part of the body, with typical symptoms including rapidly growing masses located in multiple lymph nodes. It was indicated that PARP14 is overexpressed in some subtypes of DLBCL cell lines (Camicia et al., 2013). PARP14, as a coregulator of STAT6, regulates STAT6-dependent gene expression in B cells to facilitate B-cell survival and proliferation against the adverse conditions of irradiation and growth factor deficiency, in order to avoid apoptosis (Goenka et al., 2007; Cho et al., 2009). Based on the aforementioned, classical mechanism, PARP14 can modulate the IL-4-STAT6 signaling pathway to enhance several downstream gene expressions, allowing DLBCL tumor cell survival. There are two remarkable factors (Pim-1 and Mcl-1) induced in the process of regulation to protect DLBCL against apoptosis. Moreover, as a cofactor of transcription, PARP14 can prevent cell death by inhibiting the activity of caspase-3 in the context of IL-4 induction (Cho et al., 2009).

Another typical example of the role of PARP14 in B lymphomagenesis is that IL-4 enhances glycolysis and glucose oxidation to promote the growth and proliferation of B cells. Furthermore, PARP14 accelerates lymphoma growth and, conversely, PARP14 deficiency prohibits c-Myc-induced B-lymphoid oncogenesis in a mice model. The results showed that PARP14 (mediated by IL-4) is a key component of the pro-survival signaling pathway for lymphoma (Cho et al., 2011). Therefore, PARP14 has garnered more attention as a drug target for anti-DLBCL therapy.

#### New Drug Target for Multiple Myeloma (MM)

MM, as the second most common hematologic malignancy, is characterized by the abnormal cloning and proliferation of plasma cells. Unfortunately, MM currently remains incurable (Raab et al., 2009; Rajkumar, 2011). There are no obvious initial symptoms. However, symptoms such as bone pain, frequent infections, and anemia may occur with disease progression. There are several signaling pathways involved in the occurrence and progression of human cancers. An important pathway is MAPK, which participates in the regulation of cell survival (Geest and Coffer, 2009). As a key part of this signaling pathway, the Jun N-terminal kinase (JNK) signaling pathway plays an important role in cell proliferation and apoptosis via the phosphorylation of target proteins, which is involved in tumorigenesis (Bogoyevitch and Kobe, 2006).

The JNK2-PARP14-JNK1 axis is extraordinarily vital to the fate of malignant MM cells (Barbarulo et al., 2013). JNK1 and JNK2 are two crucial members from among the JNK proteins, and the nuclear activity of JNK2 is continuously active, resulting in the protective effect and prolonged life span of MM cells, which mainly rely on the blockade of JNK1-associated apoptosis. Recent findings demonstrated that PARP14 is overexpressed in over 80% of MM cell lines. Intriguingly, JNK2 may regulate PARP14 expression in an indirect manner at the transcriptional or post-transcriptional level, which is beneficial to the survival of myeloma cells. This pro-survival function is extremely dependent on PARP14 as a mediator. As a novel downstream target of JNK2, PARP14 can facilitate MM cell survival by binding and inhibiting the kinase activity of JNK1. The suppression of JNK1 catalytic activity is achieved through the binding of JNK1 and PARP14, with the assistance of the C-terminal region of PARP14, which impedes JNK1-dependent apoptosis. One reasonable hypothesis is that the WWE domain of PARP14 related to the protein-protein interactions may be engaged in this binding process. Nevertheless, whether the PARP14 catalytic activity is indispensable in blocking JNK1 kinase activity remains unknown. Additionally, inhibition of PARP14, using the chemical inhibitor PJ34, makes MM cells much more sensitive to antimyeloma drugs (Barbarulo et al., 2013). The results may shed new light on PARP14 as an anti-MM drug target.

#### New Drug Target for Prostate Cancer (PCa)

Recent studies provide new clues about the fascinating functions of PARP14 in PCa progression (Bachmann et al., 2014). PCa is a common malignancy in men worldwide, with the tendency to metastasize from the prostate to other parts of the body. Investigations on metastatic PCa (mPCa) cell lines have revealed that PARP14, together with deltex-3-like E3 ubiquitin ligase (DTX3L) and PARP9, promoted mPCa cell survival and proliferation by forming complexes, which implies that the catalytic activity of PARP14 is indispensable for the survival of mPCa cells. To be specific, in mPCa cells, overexpression of DTX3L, PARP14, and PARP9 are constitutive in the context of interferon γ (IFNγ)-STAT1 signaling increasing. DTX3L binds to the catalytic domain of PARP9 (Takeyama et al., 2003). In mPCa cells, DTX3L can form a strong endogenous complex with PARP14 and PARP9. Interestingly, both DTX3L and PARP9 can interact with PARP14 tightly, which means that DTX3L, PARP9, and PARP14 can form complexes with each other. However, the mechanisms behind these interactions are quite distinct. Particularly, the formation of complexes between DTX3L and PARP9 or PARP14 are independent from ADP-ribosylation. On the contrary, the interaction between PARP9 and PARP14 strongly relies on mono-ADP-ribosylation. Whether the interaction between PARP9 and PARP14 is regulated by their macro domains, requires further investigation. The study indicated that the interaction between PARP10 and PARP14 is regulated by the macro domains, which would give some inspiring clues (Feijs et al., 2013; Forst et al., 2013).

Furthermore, studies also indicated that DTX3L and PARP9 regulate proliferation of mPCa cells in a STAT1-dependent manner by the inhibition of interferon regulatory factor 1 (IRF1) expression. More importantly, there is crosstalk between PARP14-STAT6 and DTX3L/PARP9-STAT1 signaling pathways to achieve the goal of mPCa cell survival (Bachmann et al., 2014). Thus, the combined inhibition of PARP14 together with other protein factors may provide an effective strategy to improve the traditional treatment of PCa.

#### New Drug Target for Hepatocellular Carcinoma (HCC)

A study concerning aerobic glycolysis (the Warburg effect) in HCC showed an amazing connection between cell survival and apoptosis (Iansante et al., 2015). HCC is not only the most common type of primary liver cancer but is also the most common cause of death from liver cirrhosis in adults. It is well-known that the rapid proliferation of cancer cells (including HCC cells) always utilizes aerobic glycolysis to provide energy for growth. In HCC and cirrhotic livers, there is an obvious up-regulation of PARP14 expression correlated with glycolytic gene expression, which clearly predicts the vital role of PARP14 in HCC cell growth and survival. Not surprisingly, the Warbrug effect is severely hampered with the knockdown of PARP14. So, what is the molecular mechanism behind this shadow?

Generally, PARP14 causes impairment of the JNK1-dependent phosphorylation of pyruvate kinase M2 (PKM2) in HCC cells. As a mediator of aerobic glycolysis in tumor cells, PKM2 can translocate to the nucleus, interacting with transcription factors such as hypoxia-inducible factor (HIF) 1α and Myc to enhance glycolytic gene expression (Yang et al., 2012). Moreover, Tyr105 residue of PKM2 can be directly phosphorylated by the tyrosine kinase fibroblast growth factor receptor-1 (FGFR1) to inactivate PKM2, resulting in a transfer of glycolytic intermediates in biosynthetic pathways (Hitosugi et al., 2009).

In HCC cells, PARP14 being a pro-survival protein can firstly impede the serine/threonine kinase activity of pro-apoptosis protein JNK1, which is an upstream regulator of PKM2. This leads to the subsequent inhibition of the nuclear function of downstream PKM2, which promotes tumor cell survival. Notably, JNK1 can directly activate PKM2 by phosphorylating at the Thr365 residue, which is crucial for the catalytic activity of PKM2. In other words, the low activity of PKM2 is suitable for the aerobic glycolysis that promotes HCC cell survival by accumulating antioxidants. In contrast, in the absence of PARP14, PKM2 is phosphorylated by active JNK1, leading to the conversion of glucose to pyruvate, which enhances cell apoptosis. In addition, the inhibition of PARP14 via inhibitor PJ34 can make HCC cells sensitive to anti-HCC drugs (Iansante et al., 2015). However, whether phosphorylation of PKM2 could cause conformational changes of the binding site for its substrates (phosphoenolpyruvate and ADP) or alteration of enzymatic function requires further investigation. The study revealed that the PARP14-JNK1-PKM2 regulatory axis plays a significant role in the aerobic glycolysis and cellular metabolism of HCC cells. Hence, the development of PARP14 inhibitors may be an effective strategy for HCC treatment.

#### PARP14 Could Be a New Drug Target for Chemosensitizer

As a traditional pharmacological theory, synthetic lethality means that an expression defect of only one gene cannot lead to cell death, but the combined expression defects of two or more genes could cause cell death. This theory has been successfully applied to molecular targeted cancer therapy.

For instance, *BRCA1* and *BRCA2* are crucial for the repair of double-stranded DNA (dsDNA) damage in homologous recombination repair pathway. Thus, *BRCA1* or *BRCA2* deficiency could lead to serious mistakes in the DNA repair process, which results in breast cancer. PARP1 performs a key role in repairing single-stranded DNA (ssDNA) damage. The application of PARP1 inhibitors to block the activity of this enzyme would cause double strand DNA breaks. In breast cancer cells with *BRCA1/2* mutations, the administration of PARP1 inhibitors would largely destroy the repair of damaged DNA by blocking the homologous recombination repair pathway, eventually leading to cell death. Thus far, the US FDA has approved four PARP inhibitors, olaparib, rucaparib, niraparib, and talazoparib. These PARP inhibitors are outstanding examples of clinically approved chemosensitizers that utilize the synthetic lethality concept (Lord and Ashworth, 2017).

Furthermore, there is evidence showing a novel role for PARP14 in homologous recombination DNA repair. Through interaction with proliferating cell nuclear antigen (PCNA), PARP14 promoted the replication of DNA even through damaged sites to maintain genomic stability. This study indicates that PARP14 inhibition and other undiscovered gene mutations beyond *BRCA1/2* deficiency might hamper the homologous recombination repair pathway, making cancer cells more susceptible to DNA lesions (Nicolae et al., 2015). In fact, the inhibition of PARP14 using inhibitor PJ34 made MM and HCC cells much more sensitive to anti-MM or anti-HCC drugs (Barbarulo et al., 2013; Iansante et al., 2015). The synthetic lethality of PARP14 inhibitors along with an identified genetic defect in the DNA repair process would make PARP14 a remarkable target for chemosensitizer design, which means the combination of PARP14 inhibitors and anticancer drugs causing DNA damage activity can amplify the cancer cell killing effect. Consequently, PARP14 could be a momentous drug target not only as an anticancer drug but also as a chemosensitizer.

### New Drug Target For Atherosclerosis

Atherosclerosis (AS), as an independent risk factor for cardiovascular diseases, is a disease characterized by the narrowing of arteries due to plaque accumulation. AS progression over time results in coronary artery disease, stroke, or kidney problems. AS is an autoimmune disease in which an immune response plays a crucial role in its occurrence and development. Studies indicated that PARP14 might regulate post-transcription in macrophages to influence the expression of tissue factor, which plays a pivotal role in AS development (Iqbal et al., 2014). AS plaques are infiltrated by a large number of T helper (Th) cells, among which Th17 cells and its cytokine IL-17 play an important role in AS formation. IL-17 promotes macrophage accumulation in plaques and the release of cytokines such as tumor necrosis factor α (TNF-α), IL-6, vascular cell adhesion molecule 1 (VCAM-1) and chemokine C-C motif ligand 5 (CCL5) (Erbel et al., 2011). Furthermore, the cytokine-activated vascular endothelial cells induce their apoptosis, which exacerbates inflammation in the vessel and promotes plaque formation (Zhu et al., 2011). Astonishingly, *in vitro*, PARP14 can regulate Th17 cell growth and differentiation as well as IL-17 secretion, to affect AS progression (Mehrotra et al., 2015). However, one recent literature illustrated that inactive PARP14 induced pro-inflammatory genes and suppressed anti-inflammatory gene expression. Moreover, a deficiency of PARP14 in hematopoietic cells worsened the inflammatory response of acute and chronic arterial damage in mice (Iwata et al., 2016). Thus, the accurate role of PARP14 in relation to AS progression is unknown and remains an urgent question to answer before taking advantage of PARP14 as a newborn drug target for AS therapy.

### New Drug Target for Allergic Inflammation

Epithelial cells of the airways and the esophagus, and keratinocytes secrete numerous cytokines in response to environmental allergens, leading to the activation of immune processes. This long-term and repeated physiological reaction causes allergic inflammatory diseases such as asthma and eosinophilic esophagitis (EoE).

Asthma is a common chronic inflammatory disease of the airways. Symptoms include wheezing, shortness of breath and coughing, which are a consequence of airway obstruction and airway hyperresponsiveness (AHR). The occurrence and progression of allergic airway diseases (AAD) is closely related to many components of the immune system, including T cells and cytokines (Broide et al., 2011). Studies on a murine model of AAD showed that *Parp*14^−/−^ mice had improved lung pathology, and the inhibition of PARP14 catalytic activity using inhibitor PJ34 attenuated the severity of lung inflammation and AHR. In addition, PARP14 promoted Th2 differentiation *in vitro* by controlling the process of STAT6 binding to *Gata3* promoter (Mehrotra et al., 2013). It was also shown that PARP14 influences the induction of IL-21, which promotes Th17 differentiation through binding target DNA (Riley et al., 2013). Cytokine IL-17A, produced by Th17, is positively correlated with airway resistance in the progression of asthma (Alcorn et al., 2010). PARP14 deficiency and inhibition of its activity by PJ34 decreased Th17 cell numbers in a model of allergic airway inflammation. Moreover, the deficiency of PARP14 in mice impaired T follicular helper (Tfh) development, which plays a key role in allergic responses (Crotty, 2014). All of these changes in Th17 and Tfh development were regulated by PARP14 through controlling the activation of STAT3 via phosphorylation (Mehrotra et al., 2015).

EoE, also known as allergic esophagitis, is an allergic inflammatory condition of the esophagus that is characterized by difficulty swallowing, food impaction, and vomiting (Blanchard et al., 2005). STAT6 regulates the expression of eotaxin-3/CCL26 to produce the accumulation of eosinophil in EoE patients (Kagami et al., 2005; Blanchard et al., 2006; Niranjan et al., 2013). Remarkably, there is an obvious increase in both PARP14 and eotaxin-3 expression, due to the binding of STAT6 with eotaxin-3 promoter in esophageal biopsies from children. The blockade of PARP14 catalytic activity, to a large extent, diminishes eotaxin-3 expression in human esophageal epithelial cells (Krishnamurthy et al., 2014).

## Inhibitors of PARP14 Domains

Currently, twenty-two structures concerning PARP14 have been released in the PDB. Among them, there are three single domain structures and nineteen complex crystal structures of PARP14 domains binding to ligands. The single domain structures include two solution structures, WWE domain (PDB ID, 1X4R) and RRM domain (PDB ID, 1X5P), and one crystal structure, macro domain 3 (PDB ID, 4ABL). Among nineteen complex crystal structures, there are five endogenous ligands such as ADP and ADP-ribose: one structure for macro domain 1 (PDB ID, 3Q6Z), one structure for macro domain 2 (PDB ID, 3Q71), two structures for macro domain 1/2 (PDB ID, 3VFQ, 4D86) and one structure for macro domain 3 (PDB ID, 4ABK). The ligands of other fourteen structures are small molecular inhibitors: one structure for macro domain 2 (PDB ID, 5O2D) and thirteen structures for the catalytic domain (PDB ID, 3GOY, 3SE2, 3SMI, 3SMJ, 4F1L, 4F1Q, 4PY4, 5LXP, 5LYH, 5NQE, 5V7T, 5V7W, 6G0W) (http://www.rcsb.org/, 2019.01.31) (Andersson et al., 2012; Wahlberg et al., 2012; Forst et al., 2013; Peng et al., 2017; Schuller et al., 2017; Upton et al., 2017; Yoneyama-Hirozane et al., 2017; Holechek et al., 2018; Moustakim et al., 2018). All the PARP14 ligands released in the PDB are summarized in Table 1. There is only one inhibitor aimed at macro domain 2 of PARP14, while thirteen inhibitors are designed to target the catalytic domain of this enzyme (Table 1). Among the released inhibitors, there are several potent candidates showing remarkable water solubility or favorable selectivity for PARP14 as sub-micromolar inhibitors (Andersson et al., 2012; Peng et al., 2017; Upton et al., 2017). In addition, the macro domains 2 and 3 of PARP14 has been proven to possess outstanding selectivity for ADP-ribose binding function, which indicates that these domains might be potential therapeutic targets for selective inhibition (Forst et al., 2013; Schuller et al., 2017; Moustakim et al., 2018; Schweiker et al., 2018). By taking advantage of the information, researchers could design novel, highly selective inhibitors targeting to the catalytic domain or macro domains of PARP14 to block its enzymatic activities. An obstacle to be aware of is the high conservation of the catalytic domain across PARP family members, which may interfere with designing selective PARP14 inhibitors that do not impact other PARP candidates. In this context, choosing macro or even WWE domains as targets can shed a new light on a possible solution (Schweiker et al., 2018).

**Table 1:** PARP14 ligands and their crystal structures available in PDB.

**PDB ID**	**Ligand**	**Target domain**	**Structure**
3GOY	3-aminobenzamide	Catalytic domain	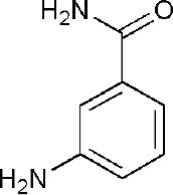
3SE2	phenanthridin-6(5H)-one	Catalytic domain	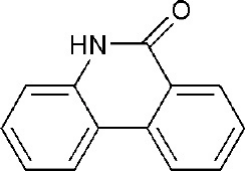
3SMI	2-{[(3-amino-1H-1,2,4-triazol-5-yl)sulfanyl]methyl}-8-methylquinazolin-4(3H)-one	Catalytic domain	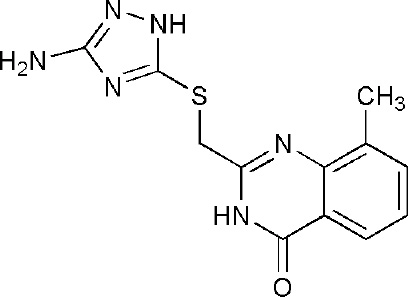
3SMJ	2-methyl-3,5,6,7-tetrahydro-4H-cyclopenta[4,5]thieno[2,3-d]pyrimidin-4-one	Catalytic domain	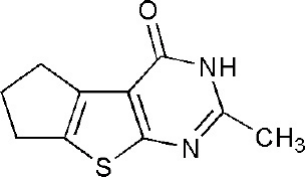
4F1L	(2Z)-4-[(3-carbamoylphenyl)amino]-4-oxobut-2-enoic acid	Catalytic domain	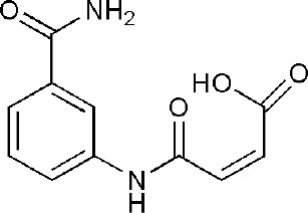
4F1Q	(2E)-4-[(3-carbamoylphenyl)amino]-4-oxobut-2-enoic acid	Catalytic domain	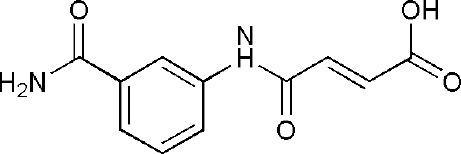
4PY4	2-({4-[(1R)-1-(dimethylamino)ethyl]phenyl}amino)-6-fluoro-1,3-benzothiazole-4-carboxamide	Catalytic domain	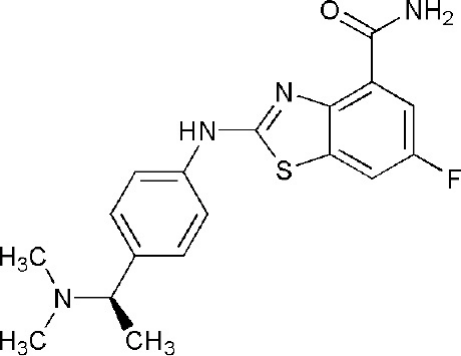
5LXP	~{N}′-(3-aminocarbonylphenyl)-~{N}-[[1-[(2~)-2-phenylpropyl]-1,2,3-triazol-4-yl]methyl]pentanediamide	Catalytic domain	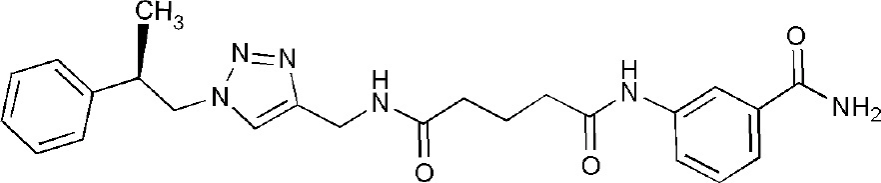
5LYH	3-[2-[4-[2-[[4-[(3-aminocarbonylphenyl)amino]-4-oxidanylidene-butanoyl]amino]ethyl]-1,2,3-triazol-1-yl]ethylsulfamoyl]benzoic acid	Catalytic domain	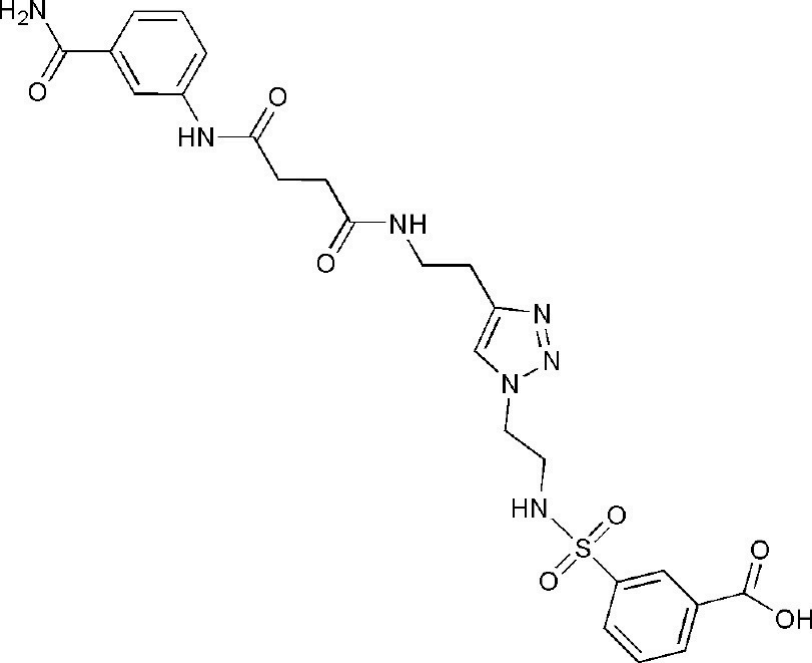
5NQE	3-[[4-[4-(4-fluorophenyl)piperazin-1-yl]-4-oxidanylidene-butanoyl]amino]benzamide	Catalytic domain	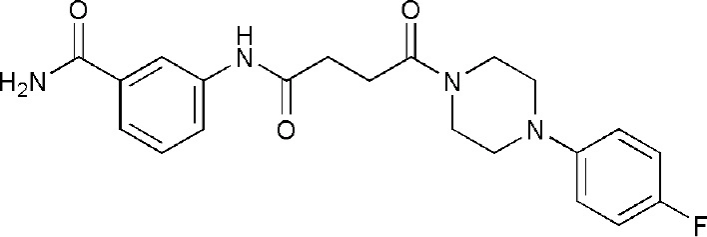
5V7T	N-{4-[4-(diphenylmethoxy)piperidin-1-yl]butyl}[1,2,4]triazolo[4,3-b]pyridazin-6-amine	Catalytic domain	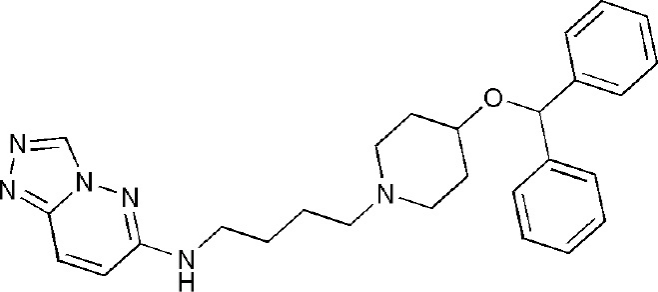
5V7W	2-{[(1-methylpiperidin-4-yl)methyl]amino}-5,6,7,8-tetrahydro[1]benzothieno[2,3-d]pyrimidin-4(3H)-one	Catalytic domain	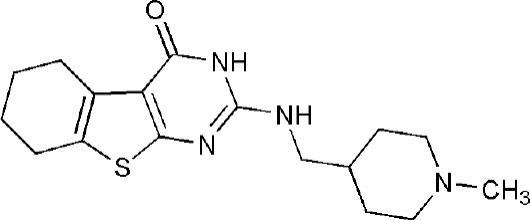
6G0W	4-[3-[4-(4-fluorophenyl)piperidin-1-yl]carbonylphenoxy]benzamide	Catalytic domain	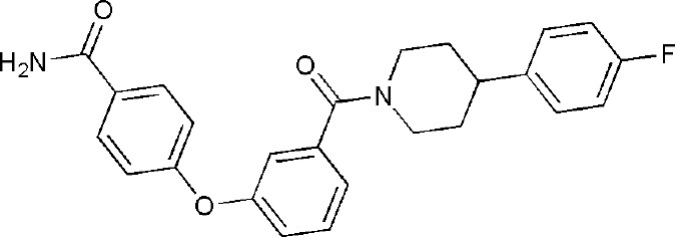
5O2D	~-[2-(9~-carbazol-1-yl)phenyl]methanesulfonamide	Macro domain 2	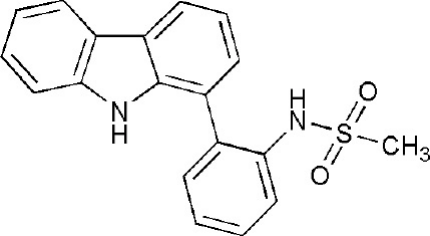
3Q6Z	adenosine-5-diphosphoribose	Macro domain 1	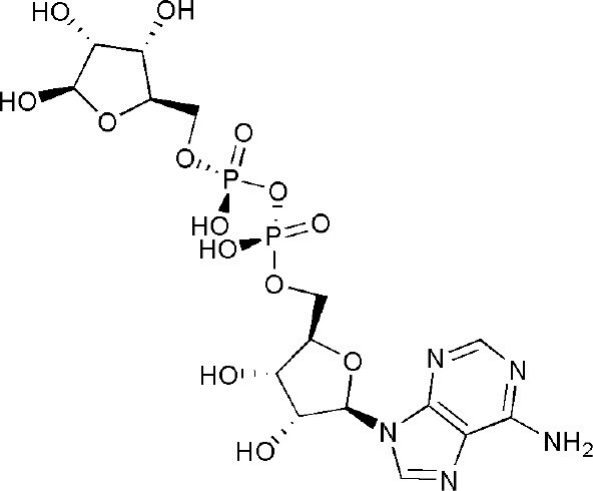
3Q71	adenosine-5-diphosphoribose	Macro domain 2	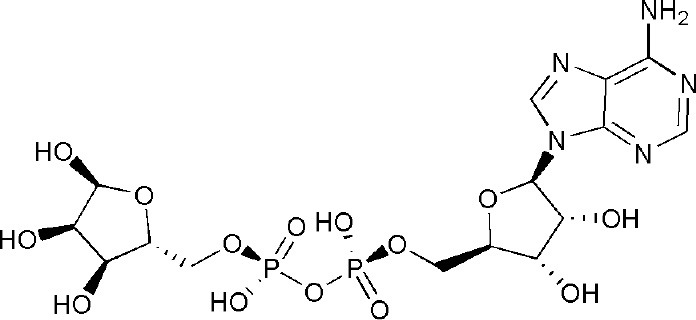
3VFQ	adenosine-5-diphosphoribose	Macro domain 1/2	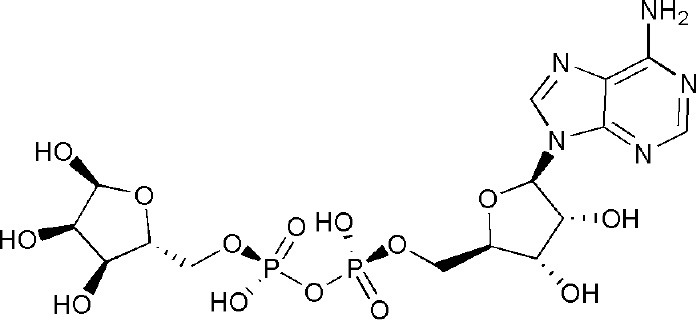
4D86	adenosine-5-diphosphate	Macro domain 1/2	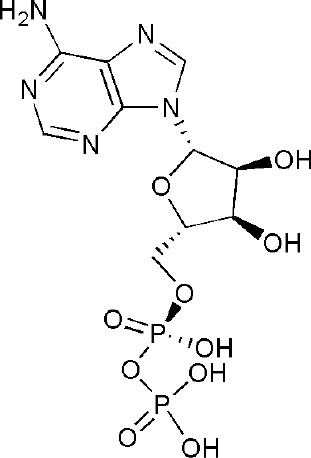
4ABK	adenosine-5-diphosphoribose	Macro domain 3	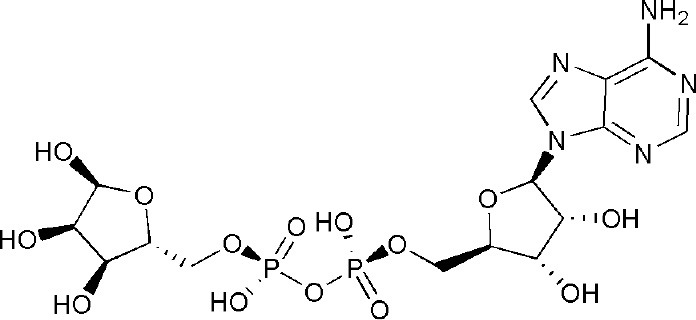

Another problem arising in treatment of diseases is the related toxicities of PARP inhibitors. Firstly, many *in vitro* studies illustrated that PARP inhibitors could severely influence chromosomal stability, thereby causing DNA injury and damage of chromosomal integrity, which can manifest dramatic genotoxicity in different cell lines (Berger et al., 2018). Therefore, one must consider the potential genotoxic effects when utilizing PARP inhibitors such as olaparib or veliparib for non-cancer indications.

Albeit no clinical trial data of PARP14 inhibitors has been reported yet, the available evidence from tests of PARP1/2 inhibitors could be reference for consideration. For instance, the associated toxicities of olaparib, niraparib, and rucaparib manifested in distinct physiological reactions in the phase III clinical trials. The typical toxic effects include hematological toxicities (anemia, neutropenia, and thrombocytopenia), gastrointestinal toxicities (nausea, constipation, vomiting, diarrhea, abdominal pain, dyspepsia, and dysgeusia), renal adverse events and fatigue. Additional unique and severe side effects pertaining to this class of drugs include neurological toxicities (headache and insomnia), respiratory toxicities (dyspnea, cough, nasopharyngitis, and upper respiratory tract infection), musculoskeletal toxicities (arthralgia and back pain), cutaneous toxicities (photosensitivity reactions, pruritus, rash, peripheral edema, and urticaria), cardiovascular toxicities (hypertension, tachycardia, and palpitations) and secondary malignancies (myelodysplastic syndrome and acute myeloid leukemia) (LaFargue et al., 2019). There is also a large amount of overlapping toxicities when PARP inhibitors are studied in doublet and triplet combination trials with antiangiogenic drugs, PI3K drugs, immunotherapy, and chemotherapy. As a result, the adverse effects of PARP inhibitors should be treated cautiously with relevant management strategies to attain maximal clinical benefit for patients (LaFargue et al., 2019).

As demonstrated by the associated adverse effects of currently approved PARP inhibitors, selectivity of inhibitors targeting PARP14 becomes more important in avoiding toxicity. This should be carefully considered in the process of designing new drugs or clinical trials concerning PARP14.

## Author Contributions

WQ and H-JW are responsible for the review writing and reference analysis. L-QC, H-JL, C-XH, and DZ focus on the reference review and analysis. LX, P-QL, and XJ devote themselves to the reference collection. H-LC commits herself to the manuscript revise and polish. All authors have contributed significantly.

### Conflict of Interest Statement

The authors declare that the research was conducted in the absence of any commercial or financial relationships that could be construed as a potential conflict of interest.
